# Eggshell debridement combined with vascularized fibular grafting for the treatment of bilateral calcaneal osteomyelitis and bone defects: a case report

**DOI:** 10.3389/fsurg.2026.1816409

**Published:** 2026-04-15

**Authors:** Guohai Li, Yinshuan Deng, Jingsheng Liu, Kang Li

**Affiliations:** 1 Department of Orthopaedics, Linxia Pukang Hospital, Linxia, China; 2 Department of Orthopaedics, Gansu Provincial Central Hospital, Lanzhou, China; 3 Department of Orthopedics, Lanzhou University Second Hospital, Lanzhou, China

**Keywords:** bone defect, calcaneus, fibular bone grafting, infection, vascular anastomosis

## Abstract

Bone defect following debridement for calcaneal osteomyelitis presents both a clinical challenge to surgeons and a significant burden to patients. A 68-year-old male patient sustained multiple injuries, including bilateral calcaneal fractures, due to a fall from height. He underwent open reduction and internal fixation (ORIF) for bilateral calcaneal fractures and left fibular fracture at an external institution. Two months postoperatively, sinus tracts developed at the bilateral incision sites, leading to a diagnosis of bilateral calcaneal infection. The patient subsequently received multiple debridement procedures and antibiotic-loaded bone cement implantation. In our management, we performed one-stage eggshell debridement and vascularized fibular grafting immediately after removing the antibiotic-loaded bone cement. At the final follow-up, radiological evaluations revealed satisfactory graft integration and stable positioning. The postoperative Maryland Foot Score was 85 for the left foot and 90 for the right foot, indicating favorable functional recovery, with the patient reporting high satisfaction. This case suggests that one-stage eggshell debridement combined with vascularized fibular grafting, performed after the removal of antibiotic-loaded bone cement following initial debridement for calcaneal infection, can provide reliable stability and achieve favorable clinical outcomes.

## Introduction

Calcaneal osteomyelitis accounts for approximately 3% to 11% of all bone infections (1, 2). The bone defects secondary to infection can occur in up to 13% of cases (3). Furthermore, literature has documented that a calcaneal defect of 13% is sufficient to cause significant impairment of ambulatory function (4). Thus, reconstructive intervention following calcaneal infection is of paramount clinical importance.

Patients with calcaneal osteomyelitis often incur substantial healthcare expenditures due to repeated surgical interventions. While emerging modalities such as 3D printing (5) and the Ilizarov technique (6, 7) have gained traction in recent years—offering merits like personalized customization and superior structural remodeling potential—their prohibitive costs, stringent hardware infrastructure requirements, and technical complexity preclude routine clinical implementation. Bone defects exceeding 6 cm are defined as large bone defects (8). Conventional iliac bone grafts are limited by insufficient graft volume; moreover, the majority of iliac grafts consist of cancellous bone, which cannot withstand weight-bearing for an extended period and exhibits suboptimal mechanical stability. Taylor et al. (9) first reported the use of vascularized free fibular grafting for the treatment of long bone defects in 1975. However, there are no high-quality reports on its application in calcaneal infections.

Some studies have identified poor blood supply to bone fragments as a risk factor for infectious nonunion of bone. Bone nonunion complicated by infection presents a dual challenge: the need to both eradicate the infection and achieve bone healing in an environment that is hostile to osteogenesis (10). Therefore, this study investigated a treatment approach for bilateral calcaneal osteomyelitis with bone defects, which involves eggshell debridement following the removal of infected calcaneal bone cement implants, combined with vascularized fibular grafting. This strategy addresses both challenges simultaneously, with the aim of achieving anatomical reconstruction of the calcaneus and maximizing the recovery of lower limb function. Additionally, the treatment process and postoperative functional outcomes were evaluated.

## Case presentation

A 68-year-old male patient sustained multiple injuries following a fall from height, including bilateral calcaneal fractures. He underwent open reduction and internal fixation (ORIF) for bilateral calcaneal fractures and left fibular fracture at an external institution. Two months postoperatively, sinus tracts developed at the bilateral incision sites, leading to a diagnosis of bilateral calcaneal infection. He subsequently received internal fixation removal, debridement, and calcium sulfate antibiotic-loaded bone cement spacer placement at another hospital, with a total of three debridement procedures performed. However, the patient continued to experience significant pain during ambulation and required bilateral axillary crutches for mobility, prompting presentation to our institution.

**Figure 1:**
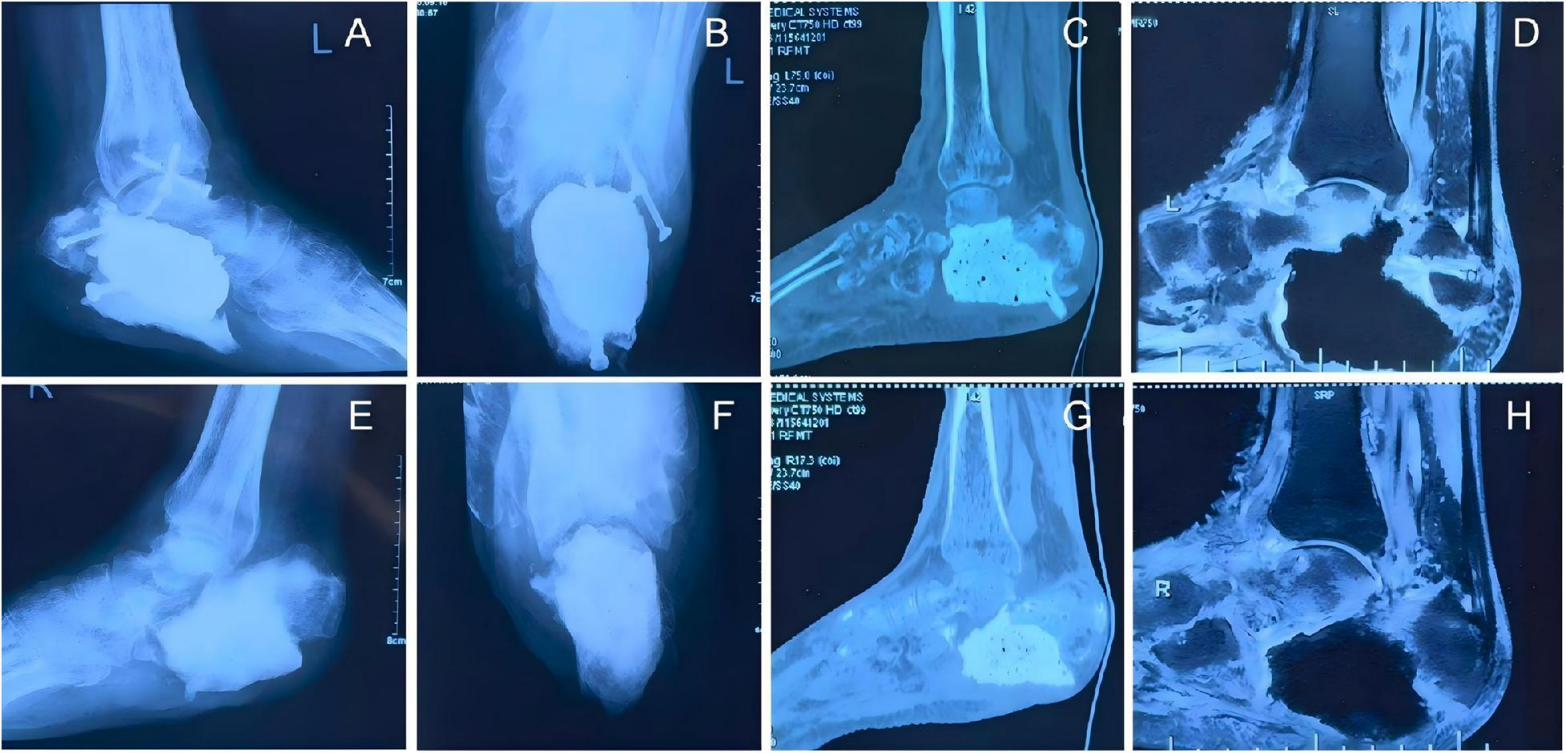
**(A–D)** shows the cement-filled status of the left calcaneus, with a large amount of cement present; **(E–H)** shows the cement status of the right calcaneus, where the amount of cement is relatively less than that on the left.

On admission, the foot surgical incisions were well-healed, and bilateral ankle joint flexion-extension range of motion was moderate. x-ray and CT scans revealed left calcaneal antibiotic-loaded bone cement spacer placement, calcaneal screw fixation, and fibular screw fixation, as well as right calcaneal antibiotic-loaded bone cement spacer placement. Magnetic resonance imaging (MRI) demonstrated pericalcaneal bone edema (Figures 1A–H), while inflammatory markers were within normal limits. Preoperative Maryland Foot Scores were 36 for the left foot and 25 for the right foot.

## Surgical technique

Prior to the current reconstruction, the local skin exhibited no erythema, swelling, warmth, or ulceration; additionally, laboratory tests for inflammatory markers (C-reactive protein, white blood cell count) yielded unremarkable results. The patient elected to undergo surgical intervention. The procedure was conducted under general anesthesia. Intravenous antibiotics (cefuroxime sodium 1.5 g) were administered 30 min preoperatively. The patient was positioned in the lateral decubitus position. No pneumatic tourniquet was employed during debridement or reconstruction.

Left-side procedure: The original left lateral L-shaped incision was re-opened to remove residual antibiotic-loaded bone cement from the calcaneus. Using a curette, 0.5 cm of bone was debrided from the cement-filled cavity (tissue samples were collected for bacterial culture and histopathological examination) until macroscopically viable bone was exposed, resulting in an “eggshell-like” residual calcaneal structure. The cavity was irrigated sequentially with normal saline. After assessing the bone defect, the ankle joint was wrapped with sterile gauze, and surgical drapes, gloves, and instruments were replaced to maintain sterility.

Make an incision along the axis connecting the fibular head and the lateral malleolus to expose the fibula and cut off a section of the fibula of the corresponding length according to the length of the bone defect. At least 6 cm of bone mass should be retained at the proximal and distal ends to maintain the structural integrity of the knee and ankle joints, and to protect the fibular artery and vein, avoiding their separation from the fibula.Following eggshell debridement, the residual posterior calcaneal segment was secured to the talus using Kirschner wires (K-wires). After assessing the defect dimensions, the fibula was transected into two segments, which were then fixed in parallel with K-wires. The K-wires were inserted from the posteroinferior to anterosuperior direction, passing through the fibular segments and anchoring to the anterior calcaneus. Given the orientation of the fibular graft, the inferior void was filled with bone cement. Axial and lateral fluoroscopic imaging confirmed restoration of the calcaneus's anatomical morphology and height.

**Figure 2:**
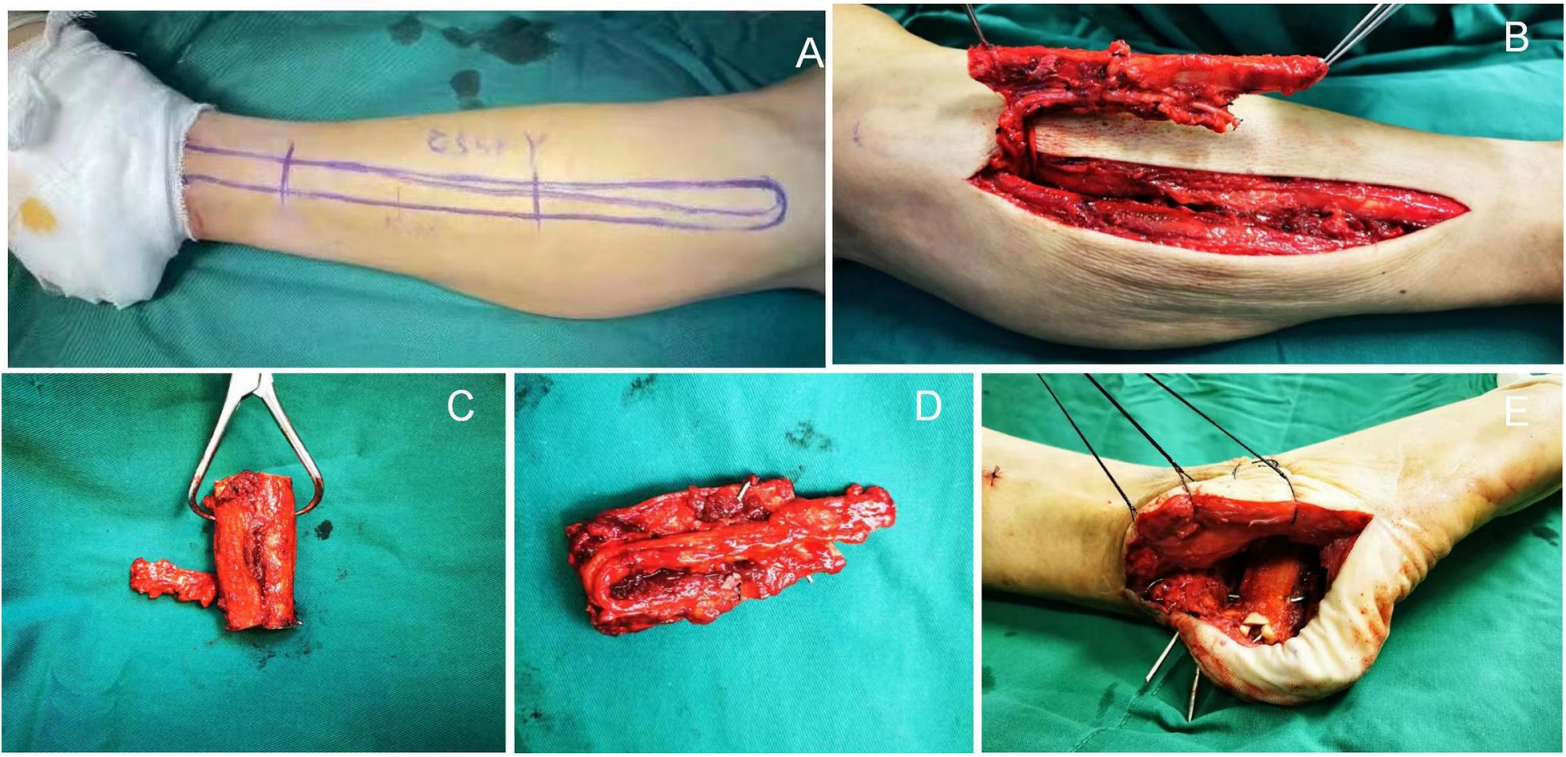
Intraoperative harvest of the left fibula. **(A)**: morphology, anatomical course of the fibula, and planned harvest site. **(B–D)**: harvested fibula and status of the vascular pedicle. **(E)**: post-implantation status of the left fibular graft. **(A,B)**: left calcaneus, showing no displacement of the fibular graft.**(C–D)**: Right calcaneus, showing no displacement of the fibular graft. All internal fixation devices were in proper position

The dorsalis pedis artery was exposed via a dorsal foot incision. A sufficiently long vascular pedicle was preserved. After fibular fixation, a skin and subcutaneous incision was made from the lateral heel extending toward the dorsal foot to create a vascular tunnel, and the vessel length was roughly planned. The dorsalis pedis artery and its accompanying vein were transected at the distal segment. Under 10× magnification (microscope), the adventitia of the dorsalis pedis artery and its accompanying vein was dissected, followed by dissection of the peroneal artery and its accompanying vein using the same technique. The vascular anastomotic sites were irrigated with 1:100 heparinized saline. End-to-end anastomosis of the dorsalis pedis artery and peroneal artery was performed with 8–0 microsurgical sutures using a point-to-point technique; the two accompanying veins were anastomosed identically. The tourniquet was released, and no active bleeding was observed at the anastomotic sites, with satisfactory pulsation noted. After a 5-minute observation period, active bleeding was confirmed at the fibular osteotomy sites. The wound was irrigated repeatedly, a drainage tube was placed, and the incision was closed in layers (Figure 2).

The surgical procedure for the right side was analogous to that of the left, with the following key modifications: Post-debridement, the posterior calcaneus retained more residual bone, which was stabilized using two 3.5-mm fully threaded screws; The fibular graft was placed horizontally, and no bone cement was used for augmentation; The implanted fibula was secured with two Kirschner wires (K-wires) in a cross-fixation configuration.

The estimated total intraoperative blood loss was approximately 100 mL. Postoperatively, the patient received routine anticoagulation, anti-infective, and antispasmodic therapies. Close monitoring of distal limb perfusion and flap vascularity was performed. The drainage tube was removed at 24 h post-surgery.

The patient was immobilized with an ankle brace in neutral position for 4 weeks postoperatively. Active hip and knee flexion-extension exercises were initiated on the second postoperative day. After 4 weeks, the ankle brace was removed, and ankle and toe functional exercises were commenced. The cross-fixation K-wires in the right calcaneus were removed at approximately 3 months postoperatively.

The patient was advised to undergo x-ray or CT follow-up at 1, 3, 6, and 12 months postoperatively to assess the osseointegration between the transplanted fibula and the calcaneus, and weight-bearing initiation was determined based on these imaging findings.

**Figure 3:**
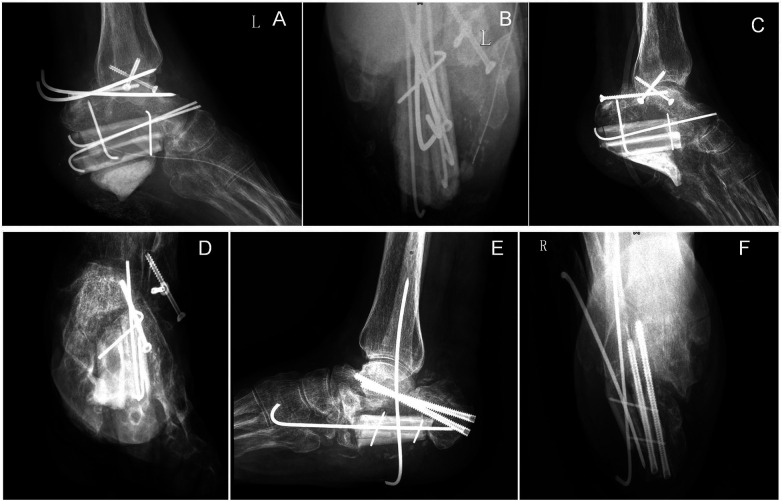
**(A,B)**: left calcaneus at 3 months postoperatively, showing a sharp protrusion at the inferior aspect of the bone cement beneath the fibular graft, which caused significant weight-bearing pain in the patient. **(C,D)**: Post-revision status: the bone cement was smoothed via open surgery using a burr, resulting in a rounded inferior contour. **(E,F)**: Right calcaneus at 3 months postoperatively.

**Figure 4:**
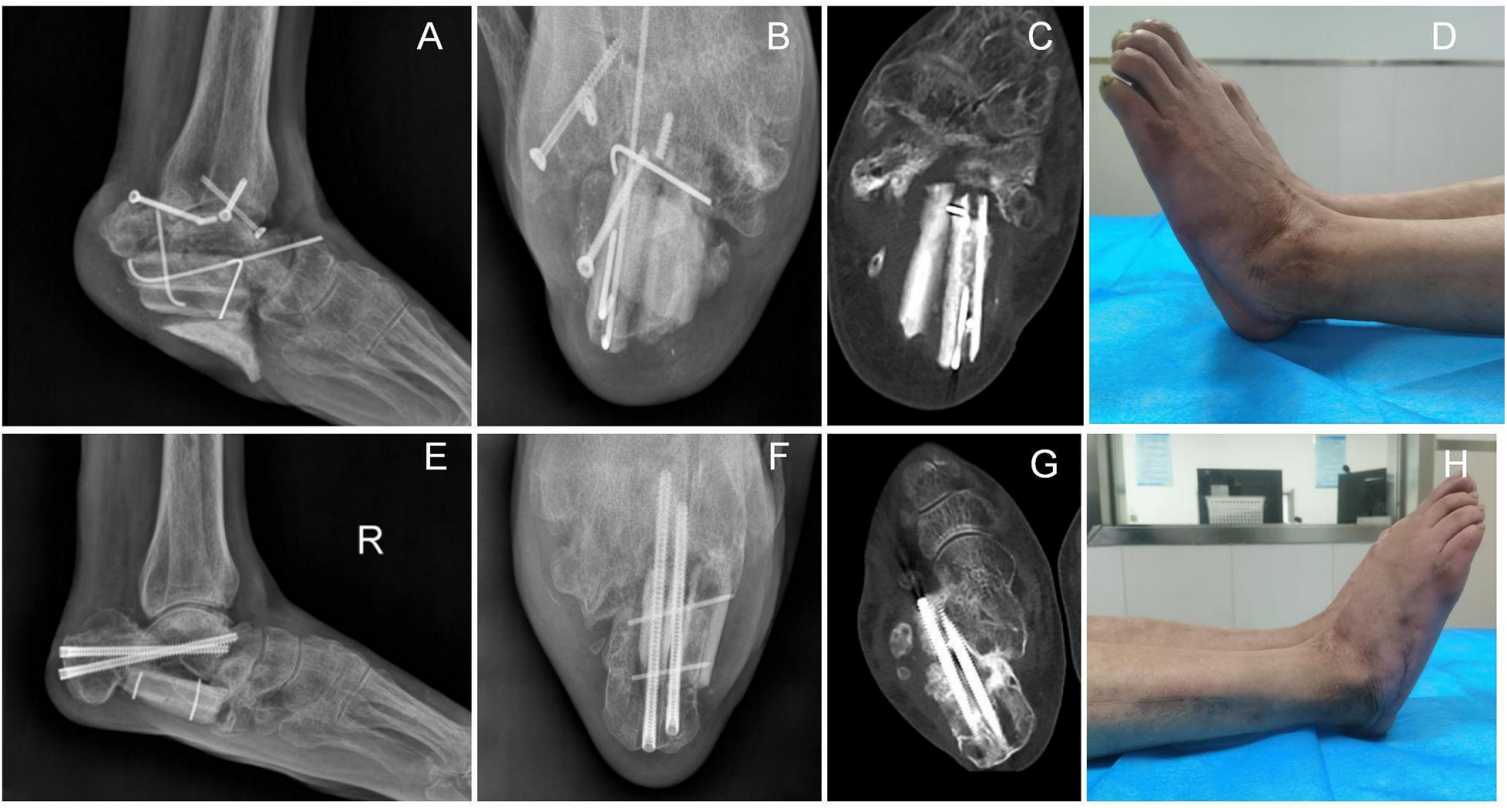
**(A–D)**: left calcaneus at 12 months postoperatively, showing well-healed incision and stable position of the fibular graft. **(E–H)**: right calcaneus at 12 months postoperatively, showing well-healed incision, osseous union, and satisfactory status of the fibular graft.

All fibular donor sites achieved primary healing, with no complications (e.g., infection, osteomyelitis) observed. During the follow-up period, the patient did not develop complications including transplanted fibular fracture, nonunion, or internal fixation failure. However, at the 3-month follow-up, a sharp protrusion was noted at the inferior aspect of the bone cement beneath the left calcaneal fibular graft, which caused significant weight-bearing pain. Consequently, a revision procedure was performed to smooth the bone cement via open surgery using a burr, resulting in a rounded inferior contour. At the final follow-up, the postoperative Maryland Foot Scores were 85 for the left foot and 90 for the right foot. The patient's ankle joint function had recovered significantly, enabling resumption of daily activities and work. The patient reported subjective satisfaction with the treatment outcome (Figures 3, 4).

## Discussion

Calcaneal osteomyelitis remains a challenging condition to manage, with surgery serving as the primary treatment modality for 97% of patients. However, clinical outcomes remain suboptimal: recurrence rates range from 20% to 35%, and below-knee amputation rates are as high as 29% (2, 11). A male predominance is observed in calcaneal osteomyelitis, which may be attributed to post-traumatic osteomyelitis accounting for 56% of all etiologies of the disease (2, 12).

Conventional surgical interventions for calcaneal osteomyelitis include radical surgical debridement, such as partial calcaneal resection, total calcaneal resection, and below-knee amputation (13, 14).In refractory cases, total calcaneal resection may be the only limb-preserving treatment option (15). However, studies have reported that the risk of infection recurrence following calcaneal resection is more than twice that of debridement alone (2). As the calcaneus is a critical weight-bearing bone of the ankle joint, every effort should be made to preserve or reconstruct it.

Given the anatomical similarity between the calcaneus and vertebral bodies, the eggshell debridement technique was first applied to calcaneal osteomyelitis by Qin et al. (16). This approach preserves the uninfected cortical bone of the calcaneus while completely resecting the internal cancellous bone, thereby achieving infection control while maintaining the calcaneal morphology—creating favorable conditions for postoperative calcaneal reconstruction and limb function recovery.

Septic nonunion represents a common complication arising during the bone healing process secondary to bone infection (17). Management of the corresponding dead space in the calcaneus is both critical and challenging. Most researchers strongly recommend calcium sulfate combined with antibiotic-impregnated therapy, as this regimen has demonstrated excellent efficacy in preventing recurrence (3, 13). While eggshell debridement maximizes the removal of cancellous bone from the cortical shell to achieve more thorough debridement (thereby reducing the risk of postoperative infection recurrence), it has limitations: the extensive debridement range may involve resection of major weight-bearing bone segments, which could compromise the planning of subsequent reconstruction procedures and prolong the time to full weight-bearing postoperatively. Additionally, bone regeneration issues persist, necessitating secondary bone grafting for calcaneal reconstruction.

Key considerations in this context include: principles governing the management of infectious bone defects (including sites beyond the calcaneus); the critical role of mechanical stability and biological stimulation in infectious reconstruction; and the understanding that successful management of infection-associated bone loss depends not merely on debridement, but on a comprehensive strategy encompassing four core pillars: thorough infection control, structural reconstruction, biological augmentation to facilitate healing, and staged or biologically guided recovery protocols (10, 17).

In the study by Down et al. (11), local antibiotic carriers were utilized intraoperatively for dead space management. The most commonly employed antibiotic carrier was a composite of calcium sulfate and hydroxyapatite, combined with gentamicin (Complex G) and/or vancomycin (Complex V).Yang et al. (4) enrolled 31 patients who underwent vacuum sealing drainage (VSD), eggshell debridement, and antibiotic-impregnated calcium sulfate implantation. Among these, 7 patients with recurrent infection and subsequent bone defects required secondary bone grafting, with calcium sulfate retained as the graft material. This combined approach has been shown to effectively control infection, facilitate soft tissue reconstruction, and promote early postoperative ambulation (11).

However, we posit that bone cement is not suitable for long-term use, as it does not support osseointegration. In the present case, bone cement combined with screws was initially considered for long-term management, but screw breakage occurred postoperatively. This complication is attributed to persistent instability and cyclic weight-bearing stress between the non-fused bone cement and calcaneus, which ultimately led to fatigue fracture of the screws.

Zhao et al. (5) employed 3D printing-assisted titanium cage fabrication combined with the Masquelet technique to reconstruct calcaneal osteomyelitis in 10 patients presenting with extensive soft tissue damage and uncontrollable defects. Among these cases, 2 patients developed infection at 2 months post the first-stage surgery, which was successfully managed via debridement and polymethylmethacrylate (PMMA) replacement. This integrated technique offers a novel strategy for limb salvage, particularly suitable for cases characterized by extensive soft tissue injury and uncontrollable defect dimensions. Regarding prevention, multiple vaccines have been recently launche (18, 19); it is anticipated that the early administration of these vaccines will help reduce the incidence of the calcaneal osteomyelitis. Rollo et al. (17) demonstrated that teriparatide, administered as an adjuvant therapy, confers significant efficacy in the management of septic nonunion, thereby potentially offering a novel therapeutic paradigm.

Wang et al. (20) introduced a modified Masquelet technique for the management of bone infection complicated by large-scale bone defects. This technique integrates the advantages of free vascularized fibular grafting and the conventional Masquelet technique, effectively circumventing their respective limitations and yielding favorable clinical outcomes. Meccariello et al. (10) posit that this approach confers enhanced biological and mechanical support for fracture healing—a perspective that aligns closely with our own rationale. The main difference between the technique we adopt and the traditional two-stage reconstruction lies in that in our eggshell debridement, the cavity wall is thoroughly scraped and structural bone grafting is applied, with very little cancellous bone from the fibula used. In contrast, the latter retains the induced membrane for bone grafting and usually uses cancellous bone. The main advantage of the bone support technique is that it provides supplementary stability and bone mass reserve through bone grafting.

We adopted eggshell debridement to minimize residual infectious foci. While this debridement approach results in a substantial bone defect, we utilized a vascularized fibula for structural reconstruction and bone grafting. The rationale for selecting the fibula is fourfold:1. The defect is excessively large, often leaving only the medial and posterosuperior cortical bone, necessitating a large volume of bone graft material; 2. The weight-bearing region requires robust osseous grafting for mechanical support; 3. The vascularized fibula maintains a viable blood supply, which enhances anti-infective capacity and promotes fracture healing; 4. The role of vascularized grafting not only as structural support, but also as a means of improving local biology in a previously infected bed.

During the harvesting of the vascularized fibula, the following considerations apply: 1. Careful dissection and protection of the common peroneal nerve are imperative; 2. At least one-third of the distal fibular bone must be preserved to prevent ankle instability caused by overly distal harvesting. Additionally, the harvested length should be determined based on the entry site of nutrient vessels into the periosteum and the number of vessels; 3. Prior to pedicle division of the fibular vessels, the tourniquet should be released to observe and confirm the integrity of blood supply to the fibular flap.

Key considerations for vascularized fibular bone grafting include: 1. The angle of fibular implantation. For the left-side fibula, which was implanted in a posterior-inferior to anterior-superior direction, a gap existed beneath the fibula. During weight-bearing, the calcaneal load shifts downward, resulting in excessive stress on the anterior-superior segment of the fibula—this predisposes to nonunion or even fatigue fracture. Thus, bone cement was utilized to fill the gap and provide supplementary mechanical support.Moreover, bone cement has strong plasticity and can be implanted according to the support requirements; 2. The vascular pedicle should be reserved with sufficient length to ensure unobstructed vascular anastomosis; 3. Given that the fibula serves as a structural bone graft, delayed weight-bearing is recommended to maintain stability for bone healing.

In the present study, favorable clinical outcomes were observed for bilateral calcaneal lesions, potentially reflecting a limb-salvage reconstruction paradigm analogous to that applied in the management of osteomyelitis-associated nonunions—extending beyond the calcaneus. Nevertheless, several methodological and clinical limitations warrant acknowledgment. The follow-up duration was restricted to approximately one year, leaving uncertainty regarding the long-term sustainability of clinical outcomes in individuals engaged in heavy manual labor.The comparability of this technique's efficacy in patients with osteoporosis remains unestablished, warranting further investigation.

Additional biomechanical studies are required to validate the stability and effectiveness of the approach.The current findings are derived from a single case; future multi-case studies are needed to replicate and generalize these results.

## Conclusion

Bilateral calcaneal bone defects secondary to osteomyelitis are relatively rare, with limited reports in the existing literature. In this case, the patient underwent first-stage eggshell debridement following polymethylmethacrylate (PMMA) removal, combined with vascularized fibular grafting. Follow-up evaluations demonstrated that the calcaneal position was stably maintained and functional recovery was favorable.

## Data Availability

The original contributions presented in the study are included in the article/Supplementary Material, further inquiries can be directed to the corresponding author.
